# Estimated Effectiveness of COVID-19 Messenger RNA Vaccination Against SARS-CoV-2 Infection Among Older Male Veterans Health Administration Enrollees, January to September 2021

**DOI:** 10.1001/jamanetworkopen.2021.38975

**Published:** 2021-12-15

**Authors:** Yinong Young-Xu, Gabrielle M. Zwain, Ethan I. Powell, Jeremy Smith

**Affiliations:** 1 White River Junction Veterans Affairs Medical Center, White River Junction, Vermont; 2 Geisel School of Medicine, Dartmouth College, Hanover, New Hampshire

## Abstract

This case-control study examines estimated COVID-19 messenger RNA vaccination effectiveness against SARS-CoV-2 infection from January to September 2021 among fully vaccinated male veterans aged 65 years or older.

## Introduction

In a recent study that estimated COVID-19 messenger RNA (mRNA) vaccine effectiveness,^1^ we found that the mRNA-1273 (Moderna) and BNT162b2 (Pfizer-BioNTech) vaccines were highly effective against SARS-CoV-2 infection before June 2021 among 1 363 180 US veterans who were vaccinated in January and February of 2021. Transmission of the SARS-CoV-2 Delta variant increased rapidly during the summer of 2021, and waning COVID-19 mRNA vaccine effectiveness has been reported.^2^ Therefore, we reexamined the estimated effectiveness of the 2 COVID-19 mRNA vaccines (mRNA-1273 and BNT162b2) during July to September 2021 among fully vaccinated male veterans aged 65 years or older.

## Methods

This case-control study was approved by the US Department of Veterans Affairs Medical Center Institutional Review Board in White River Junction, Vermont. The board deemed informed consent from millions of patients impractical and granted an exemption. The study followed the Strengthening the Reporting of Observational Studies in Epidemiology (STROBE) reporting guideline.

Electronic medical record data from the Veterans Health Administration Corporate Data Warehouse were analyzed. Based on SARS-CoV-2 variant tracking data from the Centers for Disease Control and Prevention,^3^ we divided the observation time into 3 periods: pre-Delta (before May 2021), rising Delta (May and June 2021), and high Delta (July through September 2021) when the Delta variant accounted for more than 70% of new infections in the US.^3^

A matched case-control study was conducted to estimate mRNA vaccine effectiveness against SARS-CoV-2 infection. Details of the methods used were published previously.^1^ Briefly, negative SARS-CoV-2 tests served as controls, and a maximum of 4 controls were matched to each case based on Department of Health and Human Services geographic region and testing date (within 21 days of the case testing date) because both factors are related to local disease burden, likelihood of having a positive SARS-CoV-2 test result, and vaccination status.

Conditional logistic regression was conducted with full vaccination status as the primary explanatory variable. The exposure of interest for vaccinated individuals was the time since vaccination, measured as the difference in months from the full vaccination date (≥14 days after the second dose) to the event date (a positive reverse transcription polymerase chain reaction SARS-CoV-2 test result). The regression model included indicator variables representing months since vaccination, Delta periods, and their interaction terms. The association between SARS-CoV-2 infections and time since vaccination was examined by estimating the odds ratio for each month after receipt of full vaccination, adjusting for additional variables including age and comorbid conditions (Table).

**Table.** Change in Estimated Messenger RNA Vaccine Effectiveness Against Laboratory-Confirmed SARS-CoV-2 Infections, January to September 2021

Month	Adjusted vaccine effectiveness by month from full vaccination, % (95% CI)^a^		
	Pre-Delta (January to April)	Rising Delta (May to June)	High Delta (July to September)
1	94.5 (90.7-96.7)	92.1 (87.2-95.1)	62.0 (45.6-73.5)
2	88.5 (86.1-90.5)	90.6 (87.8-92.7)	60.9 (51.5-68.4)
3	87.9 (85.9-89.5)	87.3 (80.8-91.7)	57.8 (52.5-62.5)
4	NA	86.6 (83.0-89.5)	38.3 (33.5-42.7)
5	NA	67.3 (63.2-70.9)	18.9 (13.7-23.8)
6	NA	NA	18.4 (13.3-23.3)
7	NA	NA	23.4 (17.3-29.0)
8	NA	NA	24.8 (18.8-30.4)

Abbreviation: NA, not applicable.

^a^ Male veterans aged 65 years or older with positive SARS-CoV-2 test results (cases) or negative test results (controls) were matched 1:4 on time of test and geographic region. Adjusted variables included the following: age, body mass index, cancer, congestive heart failure, chronic kidney disease, chronic obstructive pulmonary disease, diabetes, hypertension, immunocompromised status, priority level, race and ethnicity, and rurality. See eTable 1 in the Supplement in Young-Xu et al^1^ for definitions of these variables.

All tests were 2-tailed, and statistical significance was set at *P* = .05. Statistical analysis was performed using SAS version 9.4 (SAS Institute).

## Results

In this study, there were 14 238 male veterans aged 65 or older with a positive SARS-CoV-2 test result (cases) and 56 952 veterans with a negative test result (controls) (Table). The estimated pre-Delta mRNA vaccine effectiveness against any SARS-CoV-2 infection was 94.5% (95% CI, 90.7-96.7) in the first month after complete vaccination (Figure; Table) and decreased to 87.9% (95% CI, 85.9-89.5) by month 3. During the high-Delta period, the estimated vaccine effectiveness was 62.0% (95% CI, 45.6-73.5) in the first month and decreased to 57.8% (95% CI, 52.5-62.5) by month 3, similar to the pattern from the pre-Delta period. The decrease in vaccine effectiveness accelerated after month 4, reaching a low of approximately 20% in months 5 through 7.

**Figure.**
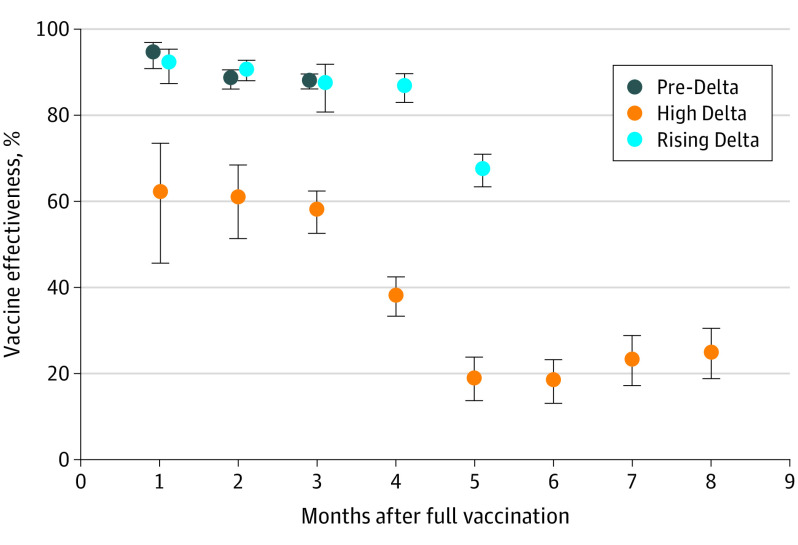
Estimated Messenger RNA Vaccine Effectiveness Against SARS-CoV-2 Infection by Delta Variant Period, January to September 2021

We estimated vaccine effectiveness by month from full vaccination for 3 months of the pre-Delta period and for 5 months during the rising-Delta period against all 8 months available during the high-Delta period (Figure; Table). Where the 95% CIs did not overlap, vaccine effectiveness was significantly different.

## Discussion

Similar to our previous findings,^1^ estimated vaccine effectiveness against SARS-CoV-2 infection was approximately 90% during the pre- and rising-Delta periods in the first 3 months after full vaccination. During the high-Delta period, we found a pattern similar to that observed in a study from Qatar,^2^ in which the estimated vaccine effectiveness against infections was significantly lower (about 60%) and the decrease in vaccine effectiveness accelerated after month 4 after full vaccination. However, the focus on older male veterans in our study could limit the generalizability of these findings.

Studies suggest that the currently approved 2-dose mRNA vaccines (mRNA-1273 and BNT162b2) seem to generate sufficient protection against COVID-19–related hospitalization despite increased transmission of the Delta variant.^2,4^ In this study, estimated vaccine effectiveness against SARS-CoV-2 infection decreased significantly to around 20% in months 5 through 7. This decrease could be attributable to both the passage of time (ie, a waning effect) and the increased transmission of the Delta variant.
